# Asynchrony induces polarization in attraction-based models of collective motion

**DOI:** 10.1098/rsos.190381

**Published:** 2019-04-24

**Authors:** Daniel Strömbom, Tasnia Hassan, W. Hunter Greis, Alice Antia

**Affiliations:** 1 Department of Mathematics, Uppsala University, Uppsala 75601, Sweden; 2 Department of Biology, Lafayette College, Easton 18042, PA, USA; 3 Department of Biosciences, College of Science, Swansea University, Swansea SA2 6PP, UK; 4 Department of Mathematics and Statistics, Carleton College, Northfield 55057, MN, USA

**Keywords:** self-propelled particles, synchrony, self-organization, flocking, collective behaviour

## Abstract

Animal groups frequently move in a highly organized manner, as represented by flocks of birds and schools of fish. Despite being an everyday occurrence, we do not fully understand how this works. In particular, what social interactions between animals give rise to the flock structures we observe? This question is often investigated using self-propelled particle models where particles represent the individual animals. These models differ in the social interactions used, individual particle properties, and various technical assumptions. One particular technical assumption relates to whether all particles update their headings and positions at exactly the same time (synchronous update) or not (asynchronous update). Here, we investigate the causal effects of this assumption in an attraction-only model and find that it has a dramatic impact. Polarized groups do not form when synchronous update is used, but are produced with asynchronous update, and this phenomenon is robust with respect to variation in particle displacements and inclusion of noise. Given that many important models have been implemented with synchronous update only, we speculate that our understanding of the social interactions on which they are based may be incomplete. Perhaps previously unobserved phenomena will emerge if other potentially more realistic update schemes are used.

## 1. Introduction

Moving animal groups such as schools of fish and flocks of birds often move in a highly coordinated fashion. How do such organized groups emerge despite the fact that each member of the group only experiences its immediate surroundings and often no leader can be identified? This question is typically investigated using self-propelled particle (SPP) models. In a typical SPP model, a number of particles move in the plane, or space, and update their headings at each time step according to a specified local interaction rule operating on the position, and/or the heading, of nearby particles. An example of a common local interaction rule is one where particles are repelled from nearby particles (repulsion), take the average heading of particles at intermediate distances (orientation), and are attracted to particles which are further away (attraction) [1,2]. A large number of models implementing various subsets of attraction, repulsion and alignment have been proposed and analysed in recent years [3,4].

SPP models have proven successful in explaining how collective motion may emerge from repeated local interactions between individuals in general settings and in specific experimental and real-world situations [3,4]. However, the use of SPP models has also attracted some criticism, both as models of real-world phenomena and in relation to how they are constructed [5–7]. Over the past decade, models have been adapted in various ways to resolve some of the issues. For example, models have included non-constant speeds [5], more realistic neighbour detection [8–10], more realistic visual system [11], leaders and shepherds [12–14], explicit environmental and social coupling [15], alignment-free interactions [16–20] and much more.

Although other specific concerns are outlined in [5–7], most that have been addressed focus on improving the models by making some aspect of the individual particles or their interactions more realistic. What about more low-level assumptions and choices? For example, regardless of how sophisticated the individuals and the social interactions between them are, all SPP models must include instructions for how to update particle headings and positions. One option is to update all particle positions and headings at exactly the same time (synchronous update), or use some type of asynchronous update scheme where particles may update their headings and positions at different times. This issue has been thoroughly investigated in related fields and shown to be important. For example, in robotics [21–24], cellular automata [25–29], coupled map lattices [30] and Ising spin systems [31]. In particular, direct comparison of asynchronous and synchronous versions of particular cellular automata show that asynchronous update tends to increase the stability of the automaton (see [28] for an overview).

Direct comparisons of this type are largely absent from the SPP model literature, and in most models, it is assumed that all particles calculate and update their headings synchronously. This assumption has been questioned by several authors and some have chosen to implement asynchronous update schemes [32–36]. These studies have revealed that implementing an asynchronous update scheme allows for some previously elusive empirical observations to be reproduced by SPP models. In particular, speed distributions in fish schools [32], interactions of a topological nature consistent with those observed in starling flocks [33], and collective motion in locusts [35] and soldier crabs [36]. However, despite these particular empirically motivated findings, systematic direct comparisons of various update schemes in standard SPP models have not been conducted, and it is still largely unknown what effects the choice of update scheme may have on models of this type. From a mathematical/computational point of view this choice may well have a dramatic effect; potentially in a way similar to other well-documented choices made in model construction like choices between discrete and continuous or spatial and non-spatial models [37].

Here we compare the effect of implementing the synchronous and a particular asynchronous update scheme in the simplest SPP model known to produce the three standard groups (polarized groups, mills and swarms), the local attraction model (LAM) [34]. If this choice has an effect on this model, it is likely to have an effect on more sophisticated models, and as much of our current understanding of collective motion in moving animal groups is based on SPP models, this would be a valuable insight.

## 2. Model and methods

**Figure 1.**
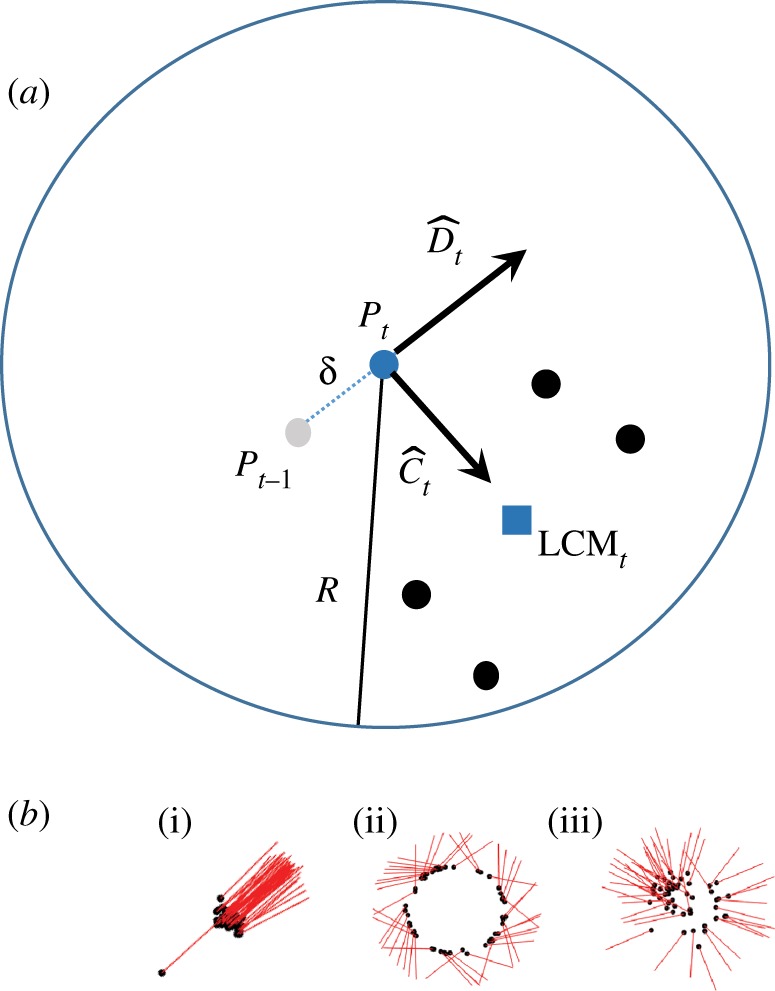
(*a*) Illustration of how a particle at position *P*_*t*_ calculates the ${\hat{C}}_{t}$ and ${\hat{D}}_{t}$ vectors in the heading update formula (equation (2.1)) on each time step. Black dots represent the neighbours of the particle and the blue square represents the LCM of the neighbours. (*b*) The groups present in the simplest version of the LAM model; (*b*(i)) Polarized group, (*b*(ii)) Mill and (*b*(iii)) Swarm. Dots represent position and rods the current heading (${\hat{D}}_{t}$). Note that the mills are undirected, i.e. particles traverse a mill in both the clockwise and counterclockwise directions.

The LAM is an SPP model in which *N* particles move at constant speed in two dimensions and interact via local attraction only [34] (figure 1*a*). On every time step, each particle calculates the position of the local centre of mass (LCM) of all particles within a distance of *R* from it (its neighbours). The new heading of particle *i* (${\bar{D}}_{t+1}^{i}$) is a linear combination of the normalized direction toward the LCM (${\hat{C}}_{t}^{i}$), its normalized current heading (${\hat{D}}_{t}^{i}$) and a normalized uniform noise vector (${\hat{E}}_{t}^{i}$).

2.1 $${\bar{D}}_{t+1}^{i}=c{\hat{C}}_{t}^{i}+{\hat{D}}_{t}^{i}+e{\hat{E}}_{t}^{i}.$$

The parameter *c* specifies the relative strength of attraction to the LCM when the relative tendency to proceed with the current heading is 1 and the parameter *e* specifies the intensity of the vectorial noise term. The particle will then move a distance of *δ* in the direction specified by ${\bar{D}}_{t+1}^{i}$. From [34], we know that different groups will form depending on if *c* is less than, approximately equal to, or larger than 1. More specifically, if *c* ≪ 1 polarized groups form (figure 1*b*(i)). If *c* ≫ 1 swarms will form (figure 1*b*(iii)). If *c* ≈ 1 undirected mills will form (figure 1*b*(ii)). At least this holds when an asynchronous update scheme is used and the noise intensity *e* = 0.

### 2.1. Synchronous and asynchronous update schemes

**Figure 2.**
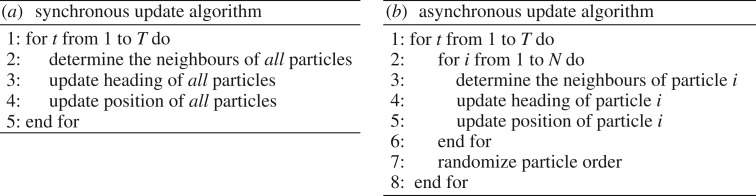
Pseudocode for the synchronous update (*a*) and the asynchronous update (*b*). For each time step *t* from 1 to the maximum simulation time *T* both algorithms update the position of each of the *N* particles, but how the three update steps (determine neighbours, update heading and update position) are carried out is different. With the synchronous update, each step is completed for all particles before the next step starts, whereas with the asynchronous update all three steps are carried out for one particle (*i*) before the first step is carried out for the next particle (*i* + 1). See electronic supplementary material, codeS2.m for the exact implementation of the asynchronous update (lines 53–92 and 148–152) and the synchronous update (lines 99–143).

The synchronous update scheme is the standard updating scheme where all particles calculate their new headings and update their positions at exactly the same time on each time step (figure 2*a*). The asynchronous update scheme chosen here is one in which all the particles update their headings and positions sequentially on each time step, and the order in which they do so is randomized from one time step to the next (figure 2*b*). This particular choice is motivated in the discussion.

### 2.2. Simulations and measures

First, we ran 100 simulations for each *c* from 0.04 to 2 in increments of 0.02 in both the synchronous case and in the asynchronous case without noise (*e* = 0). Keeping all other parameters fixed at *N* = 50, *R* = 4, *δ* = 0.5. Periodic boundary conditions were used and at the start of each simulation each particle was assigned a uniformly distributed position and heading. A simulation will terminate when the maximum time *T* is reached, or when a specific group type has been identified. In this article, *T* = 15 000 time steps, except when investigating polarized group formation in the synchronous case in which it is set to *T* = 10^8^. A simulation may terminate before the maximum time is reached if a certain group has formed early. This is determined by continuously measuring the polarization *α* and scaled size *σ* throughout the simulation and comparing them to values associated with known groups.

The polarization measures the degree to which the *N* particles are heading in the same direction and is defined by

2.2 $$\alpha =\frac{1}{N}\left|\sum_{i=1}^{N}{\hat{D}}_{i}\right|,$$

where *N* is the total number of particles and ${\hat{D}}_{i}$ is the normalized current heading of particle *i* [38]. By definition, *α* ranges from 0 to 1 and polarized groups have large *α* values and mills have small *α* values. Swarms and random configurations have intermediate *α*-values. The size measure of a group of *N* particles distributed on a square of side length *L* is given by

2.3 $$\sigma =\frac{\left(\Delta P_{x})(\Delta P_{y}\right)}{L^{2}},$$

where Δ*P*_*x*_ is the length of the range of particle *x*-coordinates and Δ*P*_*y*_ is the length of the range of particle *y*-coordinates [34]. It provides an estimate of how much of the available space the group of particles occupy. If no group formed *σ* is large, cohesive polarized groups have small *σ*, mills have a *σ* value that decreases with *c*, and swarms have very small *σ*. Combining these two measures allows us to distinguish between the three groups in figure 1*b* and the case when no group has formed. In this study, we chose *L* = 10 to ensure that at most one group is present at the end of each simulation so that the polarization and scaled size measures are well defined. If *L* is larger, multiple copies of the groups may be present (see electronic supplementary material, codeS2.m).

If over 50 consecutive time steps *σ* < 0.01, a cohesive group has formed and the *α*-values will inform us about which one it is. If over 50 consecutive time steps *α* > 0.995, a polarized group has formed, and if over 50 consecutive time steps 0.01 < *σ* < 0.25 and *α* < 0.02 a large mill has formed. If any of these three situations are detected, the simulation will terminate early. Once a simulation has terminated, either by reaching the maximum time or terminating early, we collect the mean of *α* and *σ* over the last 50 steps of the simulation, and the number of time steps it took until the simulation terminated *τ*.

We also ran a set of simulations to determine how varying *δ* and *e* affects group formation. We used *δ* = 0.001, 0.3, 1 and 2, and *e* = *c*/10, *c*/2, *c* and 5*c*, and for each *δ* and *e* value we ran 70 simulations following the simulation protocol described above.

To investigate the formation of polarized groups in more detail, we ran a set of simulations containing a mix of *N*_*a*_ asynchronously updating particles and *N* − *N*_*a*_ synchronously updating particles with *c* = 0.1. More specifically, we considered four total group sizes *N* = 10, 50, 100 and 200, and ran 100 simulations for each (*N*, *N*_*a*_)-pair with *N*_*a*_ from 0 to *N* and then calculated the average polarization as a function of proportion of asynchronously updating particles *N*_*a*_. We did this in two ways, one where the asynchronously updating particles update before the synchronously updating particles on each time step, and one where they update after the synchronously updating particles on each time step.

## 3. Results

**Figure 3.**
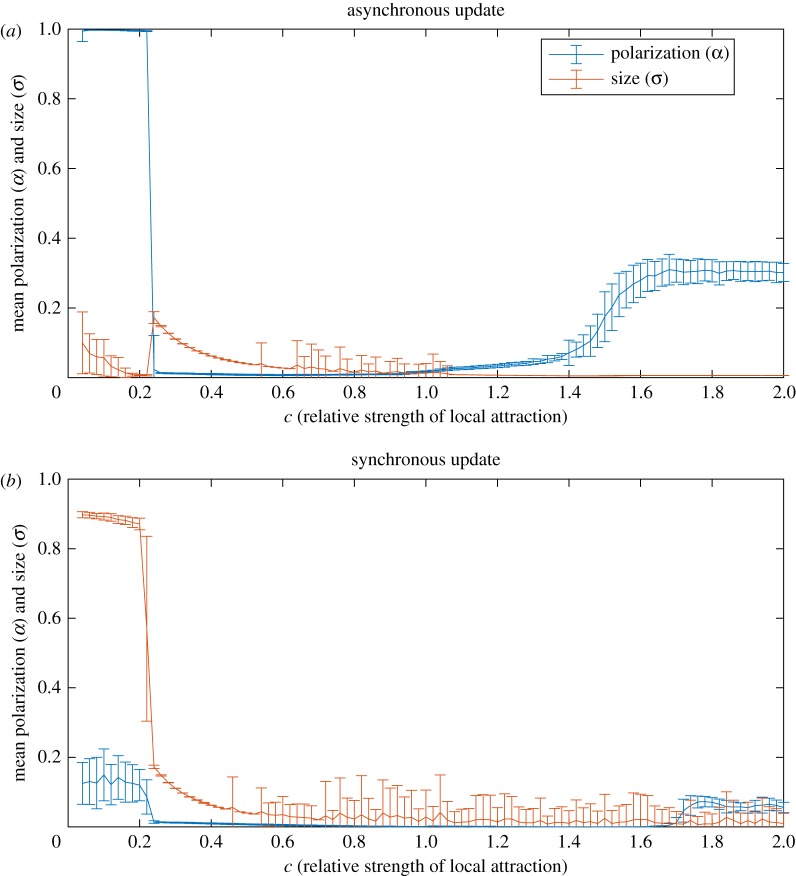
Polarization and size with asynchronous update (*a*) and synchronous update (*b*). The curves represent the mean and the bars the standard deviations over 100 simulations for each *c* ∈ [0.04, 2] in the noise-free (*e* = 0) case. In (*a*), we see that for *c* ∈ [0.04, 0.22] cohesive polarized groups form (*α* large and *σ* small), for *c* ∈ [0.22, 1.4] mills form (*α* small and *σ* small and decreasing with *c*), and finally for *c* ∈ [1.4, 2] mobile swarms form (*σ* small and *α* small to intermediate). In (*b*), we see that for *c* ∈ [0.04, 0.22] no group forms (*σ* large and *α* around the value expected if random headings), for *c* ∈ [0.22, 1.7] mills form (*α* small and *σ* small and decreasing with *c*), and finally for *c* ∈ [1.7, 2] relatively stationary swarms form (*σ* small and *α* small).

**Figure 4.**
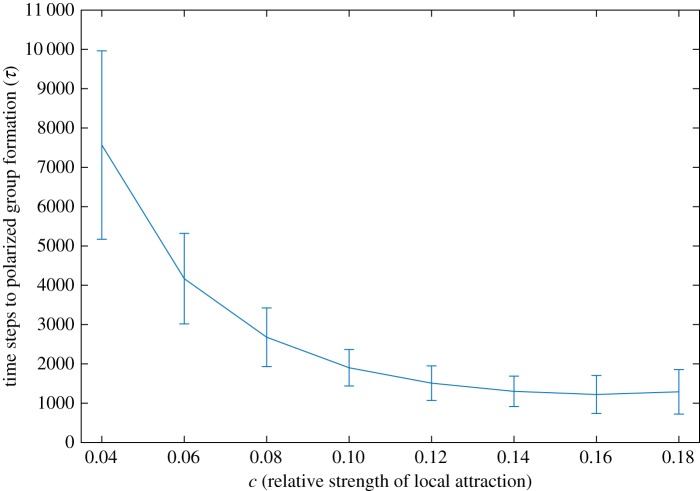
Time to cohesive polarized group formation over 100 simulations for each *c* ∈ [0.04, 0.18] with asynchronous updates. The curve represents the mean and the bars the standard deviations.

Depending on the choice of synchronous (figure 2*a*) or asynchronous update (figure 2*b*), the tendency of the model to produce polarized groups (figure 1*b*(i)) is very different. In figure 3, we see that for *c* < 0.2 there is a dramatic difference between the asynchronous update and synchronous update. In the former, we see the signature of polarized groups (large *α*, small *σ*) and in the latter, no group (small *α*, large *σ*). Figure 4 shows the time to polarized group formation for *c* from 0.04 to 0.18 over 100 simulations in the asynchronous case. We see that the time to formation decreases with increasing *c* and tends to be less than 10 000 time steps. Corresponding simulations in the synchronous case with an upper time limit of 10^8^ time steps did not produce a single polarized group. For *c* from just above 0.2–1, the asynchronous and synchronous update both produce mills and this regime appears largely unaffected by update scheme choice. For *c* larger than 1, the asynchronous and synchronous update produce qualitatively different results. In the asynchronous case, mills quickly degenerate as *c* increases above 1 and from then on partially mobile swarms are produced. In the synchronous case, mills are produced for *c* larger than 1 and only become truly degenerate and swarm-like very close to *c* = 2.

### 3.1. Influence of varying the displacement *δ*

**Figure 5.**
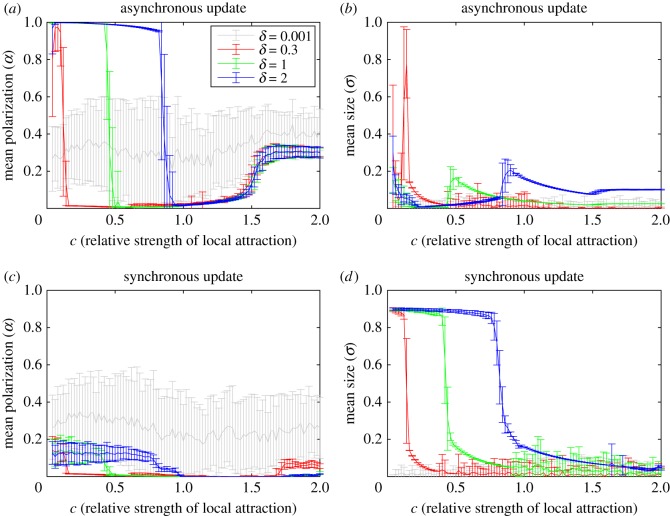
Effects of varying the displacement *δ* on group formation in the model. For *δ* = 0.001, 0.3, 1 and 2 with asynchronous update: (*a*) polarization (*b*) size, and with synchronous update: (*c*) polarization (*d*) size. The curves represent the mean and the bars the standard deviations over 70 simulations for each *c* value. Comparing the *δ* = 0.001 curves in (*a*)–(*d*), we see that both updates produce similar results; very dense groups (*σ* very small) that exhibit wildly fluctuating polarization values for all *c*. For *δ* = 0.3, 1 and 2, we see in (*a*) that the asynchronous update produces polarized groups over a range of *c* values that increases with *δ*, and by comparing (*a*) with (*b*) we see that the synchronous update produces no group over the corresponding range of *c* for each *δ*.

The effects of varying *δ* on the model behaviour are illustrated in figure 5. For *δ* = 0.001, the asynchronous and synchronous update models both produce dense swarms with erratically fluctuating polarization values for all *c* from 0.04 to 2. As *δ* increases through 0.01 both updates start producing mills and as *δ* increases beyond 0.3 the asynchronous update model starts producing polarized groups, whereas the synchronous update model starts producing no group in the corresponding range of *c*-values. This behaviour persists as *δ* increases through 1, and the upper bound on the *c* range that produces polarized groups in the asynchronous case, and no group in the synchronous case, increases with *δ*. For example, polarized groups are produced up until *c* ≈ 0.2 when *δ* = 0.5 (figure 3) and up until *c* ≈ 0.4 when *δ* = 1, and *c* ≈ 0.8 when *δ* = 2.

### 3.2. Influence of varying the noise intensity *e*

**Figure 6.**
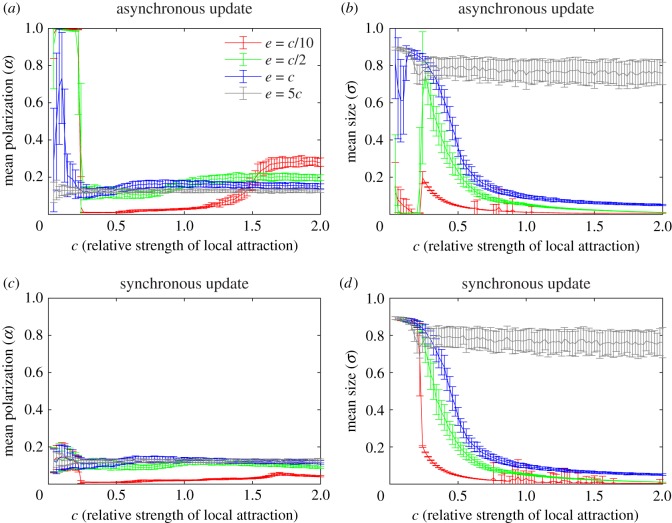
Effects of varying the noise intensity *e* on group formation in the model. For *e* = *c*/10, *c*/2, *c* and 5*c* with asynchronous update: (*a*) polarization (*b*) size, and with synchronous update: (*c*) polarization (*d*) size. The curves represent the mean and the bars the standard deviations over 70 simulations for each *c* value. Comparing the *e* = *c*/10 curves in (*a*) and (*b*) with figure 3*a*, and the curves in (*c*) and (*d*) with figure 3*b* we see that the differences in both cases are small. Combining (*a*) and (*b*), we see that cohesive polarized groups still form for *e* = *c*/2, but that the mill and swarm regimes are severely affected and now produce something that can be described as mill-swarm hybrids whose size decreases with *c*. By comparing (*a*) and (*b*) with (*c*) and (*d*), we see that the impact of increasing the noise intensity has a similar impact on the mill and swarm regimes of both models and for *e* ≤ *c* they produce essentially the same type groups in these regimes.

The effects of varying *e* on the model behaviour are illustrated in figure 6. For noise intensities up to *c*/10, the behaviour of the asynchronous and synchronous update models are largely unaffected (cf. figure 3). At *e* = *c*/2 the behaviour of the models for small *c*, i.e. asynchronous update produces polarized groups and synchronous update produces no groups, is largely unaffected. However, for *e* = *c*/2 the mill and swarm regimes are strongly affected in both cases, and from this noise intensity on they will produce similar types of groups in these regimes. Up until *e* = *c* mills and swarms are still produced, but as *e* increases beyond this value they start to degenerate, and at *e* = 5*c* no cohesive group will form. We also note that while cohesive polarized groups do not form when *e* = *c*, we observe a high degree of polarization (more than 0.7) for certain *c* values with the asynchronous update.

### 3.3. Influence of varying the proportion of asynchronously updating particles *N*_*a*_

**Figure 7.**
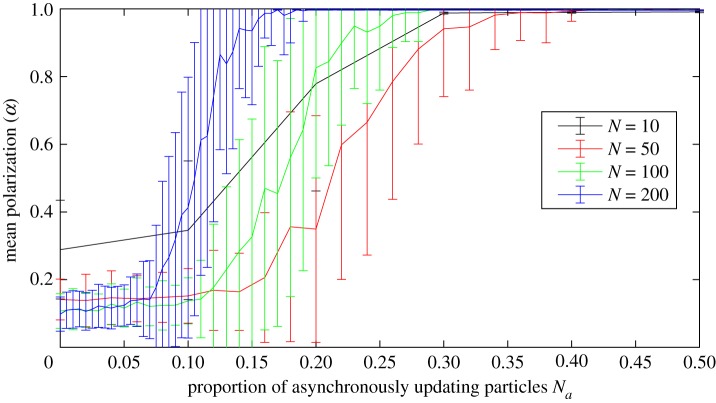
Mean polarization of groups of size *N* = 10, 50, 100 and 200 as a function of the proportion of asynchronously updating particles *N*_*a*_. The curves represent the mean and the bars the standard deviations over 100 simulations for each value of *N*_*a*_. We see that, except for the *N* = 10 case, the proportion required for consistent complete polarization decreases with group size; larger than 0.4 for *N* = 50, 0.3 for *N* = 100 and 0.2 for *N* = 200.

We established that the production of polarized groups does not require complete asynchrony in updates. Rather a certain proportion of the particles must update asynchronously for polarized groups to form. Figure 7 shows the mean polarization (*α*) over 100 simulations as a function of proportion of asynchronously updating particles for groups of sizes 10, 50, 100 and 200 and we observe that the proportion of asynchronously updating particles required for consistent polarized group formation decreases with *N* if we exclude the *N* = 10 case. In particular, polarized groups are consistently produced when the proportion of asynchronously updating particles is larger than 0.4 for *N* = 50, 0.3 for *N* = 100 and 0.2 for *N* = 200. We also found that whether the asynchronous update was before or after the synchronous has no discernible effect on this phenomenon. Therefore, we only include the result from when the asynchronous update was first in figure 7 (see electronic supplementary material, figure S1 for both).

## 4. Discussion

We have shown that the choice between asynchronous and synchronous update has a dramatic effect on group formation in the LAM. In particular, the formation of polarized groups is inhibited in the synchronous case (figure 3). Since many influential models of collective motion exclusively use synchronous updating, further analysis of these models may reveal previously unseen groups and collective phenomena which add to the asynchrony-induced speed distribution and topological-like interactions described in [32,33]. How a model will behave under the local interaction rules alone when other potentially more realistic update schemes are used is largely unknown. Perhaps properties that are rare or non-existent in groups generated by standard SPP models, for example, the multistability and transition behaviour reported in [39], are sensitive to choice of updating scheme. In particular, it appears that the synchronous update may calm the system down, as exemplified by the swarm phase in our study. In the asynchronous case, swarms are more mobile, as indicated by high polarization values for *c* > 1.4 in figure 3*a*. By contrast, in the synchronous case the swarms are almost stationary, exhibiting very low polarization for *c* > 1.7 in figure 3*b*. This is a stark contrast to the effects of synchrony and asynchrony on cellular automata described in [28] and on the coupled map lattice in [30] where it was reported that replacing synchronous update with asynchronous updates acts to stabilize the local dynamics.

In addition, our study establishes that attraction in combination with asynchrony in heading update alone induces polarized collective motion, adding to the growing literature on alignment-free models capable of producing polarized groups in two and three dimensions [16–20]. Work on models of this type is motivated, in part, by the need to explain how polarized collective motion emerges in schools of fish where no alignment responses can be detected [40,41]. These alignment-free models are attraction–repulsion models and the cause of their polarization inducing capacity is likely to be an interplay between attractive and repulsive forces, often in combination with some known polarization inducing mechanism such as asymmetric interactions (e.g. blind zones). And while these models may be preferable in some cases, we believe that there are other situations where using asynchrony in update (in combination with attraction) may be more appropriate. For example, in situations where it is known that the individuals are updating in an asynchronous manner, e.g. guppies exhibiting burst-and-glide swimming [42,43] and locusts exhibiting pause-and-go motion [44,45].

We also establish that polarized groups reliably form in simulations with a mix of asynchronously and synchronously updating particles, and that the proportion of asynchronously updating particles required decreases with the total number of particles for *N* > 10 (figure 7). This is potentially important because it shows that if a small proportion of individuals are updating asynchronously, even if these individuals are constantly changing, the group is able to agree on a common heading while interacting only via attraction. Suggesting that perhaps collective migration phenomena, typically explained by the existence of a small proportion of informed individuals within groups where individuals have an explicit tendency to align their headings [12,46] may be explained without this tendency to align. Instead the common migration heading may emerge from attractive interactions alone in combination with asynchronous updates. This would be a particularly useful mechanism for explaining collective migration in species of fish where explicit alignment responses could not be detected [40,41].

We also show that including noise (figure 6) and varying *δ* (figure 5) does not prevent polarized groups from forming when asynchronous update is used and does not enable polarized groups to form with synchronous update. The variation of *δ* study also establishes that the lower limit on *c* for mill formation (i.e. upper limit on *c* for polarized group formation/no group) appear unaffected by the choice of asynchronous and synchronous update. In [34], a simple heuristic was used to derive an approximate lower limit on *c* for mill formation in terms of *R* and *δ* [34, eq. 7] and shown to approximate the polarized group-mill boundary well via simulations [34, fig. 3]. In terms of the notation used in the current manuscript, the approximate lower limit on *c* for mill formation from [34] becomes 2*δ*/*R*, and for *δ* = 0.3, 1 and 2 this gives 0.15, 0.5 and 1, which are close to the transitions regions we observe in figure 5 in both the synchronous and asynchronous case. While this does not explain why polarized groups form in the asynchronous case and not in the synchronous case it does intuitively help explain why the range of *c* over which polarized groups/no group form increases with *δ*. Namely, because the range of *c* over which mills form decreases with *δ* which permits other groups, or no group, to form where mills used to form. The study of varying *δ* also suggests that the smallest *c* value for which polarized groups form in the asynchronous case may be dependent on *δ*. In figure 5*a*, we see that at *c* = 0.04 the mean polarization differs between *δ* = 0.3, 1 and 2. In particular, the mean polarization for *δ* = 0.3 is approximately 0.5, whereas for *δ* = 1 it is approximately 1. At present, we do not have an explanation for this phenomenon and future work is planned to address it via a thorough study of the *c* → 0 limit.

Despite being criticized, the synchronous update still seems to be the default choice in SPP model construction. We speculate that there are many reasons for this. Most well-known models were originally presented in that way, and it is more straightforward to obtain a continuum approximation of the model and thus make other analysis tools available. In addition, if one decides to use asynchronous updates, which particular scheme should one choose? The asynchronous update scheme used here, and in part in [34], was chosen mainly because it has the same update rate at the time step level as the synchronous update against which it was compared, the randomization of update sequence between time steps prevents artefacts arising from a strict persistent update order, and it was straightforward to implement in a way that allowed for a mix of asynchronously and synchronously updating particles. However, other asynchronous update schemes have been implemented [32,33,36,45–47] and these may be more suitable in some situations. In particular, we note that our asynchronous update differs significantly from the asynchronous update employed in [32,33,46], which is based on stochastic ‘neighbour picking’. Where the asynchrony is introduced via probabilistic selection of individuals to update within each time step (equal probability with replacement) and probabilistic interaction partner selection for each individual chosen to update. This differs from the asynchronous update scheme presented here where all particles will update in sequential random order on each time step and interact equally with all neighbours in the interaction zone. While the former approach, and the stochastic multiple-choice action approaches employed in [36,45], is more appropriate for studying the combined effects of noise at the level of individual behaviours/interactions and stochastic asynchronous update, our approach complements it by isolating update order-related effects via the use of deterministic interactions.

It should be emphasized that we are not claiming that the asynchronous update scheme used here is more realistic than the synchronous one against which it was compared. We do claim that this choice in itself may be critically important in the study of collective motion via SPP models. Perhaps, in some situations, it may be as important as the form of the social interaction rule itself, as in our example presented here. Therefore, we suggest that in future theoretical studies of minimal SPP models a variety of different update schemes be explored, presented and compared. In modelling specific experiments, or observations, we suggest using pilot data to estimate the update distribution and use that to inform the update choice selection. It would be very unfortunate if a carefully designed model that has the capacity to reproduce key properties observed in a specific experiment is abandoned, or made more complicated by adding more social interactions or constraints, because an underlying assumption like this one was overlooked. Our suggestions are in line with the programmes proposed in [5–7], and we believe that our work represents a concrete example illustrating the importance of continuing to work in the directions set out by these authors.

## Supplementary Material

Effects of updating the synchronous or asynchronous particles first on polarised group formation

Reviewer comments

## Supplementary Material

Animator for the local attraction model

## Data accessibility

All code used to obtain the results presented in this manuscript may be downloaded from https://github.com/danielstrombom/Asynchrony.
