# Supplementary material for: Asynchrony induces polarization in attraction-based models of collective motion
Source: R Soc Open Sci. 2019 Apr 24;6(4):190381. doi: 10.1098/rsos.190381 (PMC6502356; doi:10.1098/rsos.190381)
Supplement: Reviewer comments [file rsos190381_review_history.pdf]

THE ROYAL SOCIETY
PUBLISHING

ROYAL SOCIETY
OPEN SCIENCE

Asynchrony induces polarization in attraction-based models of collective motion

Daniel Strömbom, Tasnia Hassan, W. Hunter Greis and Alice Antia

Article citation details
R. Soc. open sci. **6**: 190381.
http://dx.doi.org/10.1098/rsos.190381

Review timeline

Original submission:	4 March 2019
Final acceptance:	15 March 2019

Note: Reports are unedited and appear as submitted by the referee. The review history appears in chronological order.

Note: This manuscript was transferred from another Royal Society journal with peer review.

Review History

Decision letter (RSOS-190381.R0)

15-Mar-2019

Dear Mr Strömbom:

It is a pleasure to accept your manuscript entitled "Asynchrony induces polarisation in attraction based models of collective motion" in its current form for publication in Royal Society Open Science. The editor is satisfied with how you have addressed the previous reviewers' comments and has recommended that the manuscript can be accepted without further peer review.

Thank you for your fine contribution. On behalf of the Editors of Royal Society Open Science, we look forward to your continued contributions to the Journal.

Kind regards,
Royal Society Open Science Editorial Office
Royal Society Open Science
openscience@royalsociety.org

Reports © 2019 The Reviewers; Decision Letters © 2019 The Reviewers and Editors; Responses © 2019 The Reviewers, Editors and Authors. Published by the Royal Society under the terms of the Creative Commons Attribution License http://creativecommons.org/licenses/by/4.0/, which permits unrestricted use, provided the original author and source are credited

on behalf of Professor Andreas Kyprianou (Associate Editor) and Professor Kevin Padian (Subject Editor).

Follow Royal Society Publishing on Twitter: @RSocPublishing
Follow Royal Society Publishing on Facebook:
https://www.facebook.com/RoyalSocietyPublishing.FanPage/
Read Royal Society Publishing's blog: https://blogs.royalsociety.org/publishing/
